# Healthcare provider and parent perceptions of newborn care and referral pathways in three hospitals in western Kenya; a formative study

**DOI:** 10.3389/fped.2025.1454756

**Published:** 2025-04-11

**Authors:** Charlotte E. Warren, Irene Namai, Florence Thungu Maloba, Harriet Ogalo, Bernard Olayo, Michel Rochat, Klaus Schönenberger, Silvan Suter, Adriane Martin Hilber

**Affiliations:** ^1^ Swiss Centre for International Health, Swiss Tropical and Public Health Institute (Swiss TPH), Basel, Switzerland; ^2^ Center for Public Health and Development, Nairobi, Kenya; ^3^ École Polytechnique Fédérale de Lausanne, EssentialTech Centre, Lausanne, Switzerland; ^4^ University of Basel, Basel, Switzerland

**Keywords:** small and sick newborn, hypothermia, KMC, referral, incubator, Kenya

## Abstract

**Background:**

The common causes of the 1.1 million newborn deaths in sub–Saharan Africa are birth asphyxia and trauma, severe infections, and complications of prematurity. Hypothermia is also a major threat to newborn survival. Three-quarters of newborn deaths could be prevented with essential equipment, skilled neonatal health workers, and a safe neonatal transport referral system. Following a review of the challenges and opportunities in caring for sick newborns, a university department (that develops sustainable and scalable solutions to address unmet needs in low-income settings) is developing an innovative newborn incubator and care solution. As part of a co-design collaborative process between the incubator developers and users, this paper explores the experiences of providers and parents of hospitalized newborns in Kenya.

**Methods:**

A qualitative design: in-depth interviews with 19 healthcare providers working in maternity unit, newborn unit, or pediatric ward; interviews with 11 parents/caregivers of hospitalized sick newborn and very young infants (0–60 days), and ethnographic observations conducted in three hospitals in Western Kenya. Data collectors experienced in qualitative methods and newborn health were trained on study topics, interview guides, and research ethics. Interviews were audio-recorded, transcribed verbatim and translated into English. Data were analyzed using NVivo 11 qualitative software.

**Results:**

The findings are presented around four themes: (1) facility infrastructure and medical supplies, devices for newborn care, and equipment maintenance; (2) characteristics of transfers/referrals of newborns between hospitals- healthcare provider views; (3) healthcare providers’ reports on caring for newborns, provider, skills, and competency in newborn care; and (4) Parents’ experiences during transfer and hospitalization of their small or sickcaring for a hospitalized baby.

**Conclusion:**

Hypothermia continues to be a problem for newborns, especially in primary healthcare settings and transfers between facilities. Potential interventions include a review of provider newborn skills and updates, including Kangaroo Mother Care, addressed through existing mentoring programs. Essential thermal equipment is also required to support quality care of small and sick newborns, including for inter and intra-facility transfers. An increased focus on providing quality thermal care of small and sick newborns is warranted.

## Background

In 2020, over 35 million or a quarter of babies born alive experienced at least one of three critical vulnerabilities – born too soon (before 37 weeks gestation), born too small (birth weight below 10th centile) or born with low birth weight (less than 2,500 g) (1). The majority of 14 million preterm births are at the highest risk of adverse neonatal outcomes and occur in low- and middle-income countries (LMICs) in Asia and sub-Saharan Africa (SSA) (2–7). Up to 75% of the 1.1 million newborn deaths each year in SSA could be prevented with essential equipment, skilled neonatal health workers, and a safe neonatal transport referral system (8). Sub optimal pre-transport and intra-transport care are associated with early neonatal morbidity and mortality due to hypothermia (9, 10). Although hypothermia is rarely the main cause of death around 40% of deaths due to hypothermia are preventable by simple and appropriate thermal management (11, 12).

One critical intervention that improves the survival of small vulnerable newborns, including the prevention of hypothermia, is immediate kangaroo mother care (KMC), which involves prolonged skin-to-skin contact with the mother and frequent and exclusive breastfeeding, started as soon as a preterm or low birthweight baby is born (13). However, there is limited provider knowledge to support the mother-baby dyad to take part in immediate and continued KMC until the baby has stabilized and can go home. Moreover, not all women are able to or interested in committing to provide KMC for their babies. When a woman cannot provide KMC, alternative solutions are needed to create a controlled and safe environment to maintain a stable temperature (between 30°C and 37°C) for babies born before 37 weeks gestation and who weigh less than 2,000 g. These solutions must be accessible, effective, and designed to meet the specific needs of low resource settings (14, 15).

In Kenya, the national neonatal mortality rate declined from 33 per 1,000 live births in 2003 to 21 per 1,000 live births in 2022. Neonatal deaths account for 66% of infant deaths and 51% of under-5 deathsand access to quality neonatal care remains variable (16). Furthermore, guidelines for transferring babies between primary health care (PHC) level and hospital level are not always followed due to sub optimal resources, and lack of skilled staff. Health workers in PHC settings transfer newborns without pre-transfer stabilization, or staff to escort the newborn, and have limited safe transport options. Most newborns travel in their mothers/caregivers' arms by car, taxi, motorcycle or on foot. Hardly any newborns arrive at a higher-level facility in an ambulance (11, 14).

This paper explores the experiences challenges and opportunities of healthcare providers and parents in caring for small and sick newborns in three county hospitals in Western Kenya. This formative study includes understanding the referral pathway for newborns from PHC to hospitals for further care as well as suggestions for improving thermal care interventions for babies in a low-income setting.

## Methods

### Study setting

In general, in Kenya, sick babies born in hospitals with respiratory distress syndrome, low birth weight, birth asphyxia, and congenital anomalies, among other complications are reviewed by a pediatrician/neonatologist and admitted to the newborn unit (NBU). Babies referred from first-level referral hospitals (level 4), PHC facilities, and those born before arrival or at home within 24 h of birth are reviewed in the emergency department and referred directly to the NBU while the mother is admitted to the postnatal ward. Newborns received more than 24 h after delivery and sick young infants (29–59 days old) are admitted to the pediatric unit which may or may not have a specific area for newborns and sick young infants (15).

The study took place in three county referral hospitals (level 5) in Homa Bay, Siaya and Vihiga counties that are similar socially, politically, and economically and are adjacent to Lake Victoriain Western Kenya. Data were collectedin February and March 2023.

This formative study adopted a qualitative design that captures in-depth interviews (IDIs) with healthcare providers working in NBUs, narratives of women with a preterm baby hospitalized in a NBU, and ethnographic observations. Eligible healthcare providers included anyone working in the unit on the day of the visit who were over 18 years and have some form of clinical training (nurses, midwives, clinical officers, or doctors). They were purposively selected and recruited from maternity, the NBU or pediatric ward in three county referral-hospitals in Homa Bay, Siaya and Vihiga Counties. Eligible parents include adults who are 18 years of age or emancipated minors, defined as those who are over 15 years of age, are married, and/or have a child. The rationale for inclusion of emancipated minors is that they and their newborns are at an elevated risk of not receiving appropriate care. Open-ended questions administered to new mothers were related to their pregnancy, delivery, and postnatal experiences including caring for their hospitalized newborn using a structured guide.

Researchers worked closely with the hospital/ward management to identify healthcare providers and parents for interviews. Provider interviews took place at their convenience, always prioritizing the needs of newborns. For example, some provider appointments were rescheduled so they could respond to emergencies, such as resuscitation of a newborn. Healthcare providers helped to identify parents who met the eligibility criteria for the interviews. For example, we did not interview mothers exhibiting symptoms of depression, or those who had only been in hospital with their baby for just a day or so. All interviews took place in private places.

Ethnographic observations also took place to define and describe the facility culture and practice norms and readiness to provide newborn care including for small and sick babies. One researcher spent a day observing movement (patient flow and provider movement and interactions) around the maternity, NBU, and pediatric units, including comings and goings of mothers during feeding times, healthcare providers' and students' interactions with each other and the mothers and during ward rounds.

Data collectors experienced in qualitative methods and newborn health were trained on study topics, interview guides, and research ethics. Data collectors had no prior established relationship with study participants. After obtaining written informed consent from participants, data collectors conducted interviews in English, Dholuo, or Kiswahili depending on each participant's preference. Interviews were audio-recorded, transcribed verbatim and translated into English. Oriented in grounded theory, following an initial reading of the transcripts, a code structure was inductively developed, discussed, and applied to the data using the NVivo 11 qualitative software. Memos written while coding the data allowed researchers to describe similarities and differences in both healthcare providers and parents's perspectives on newborn pathways to care and the potential of an accessible, contextappropriate intervention or device to support the transport of newborns between facilities. Through a deliberative process, researchers further grouped codes (first, and second order themes grouped into four main themes (see below). Researchers received ethical approval from the MASENO University Ethical Review Committee (Protocol # 01178/22), and NACOSTI – National Commission for Science, Technology & Innovation. The medical superintendents for each hospital also gave permission to conduct data collection.

## Results

### Healthcare provider and parent experiences with hospitalized newborns

This study aimed to explore healthcare provider and parent experiences of newborn care and referral in three county hospitals. Nineteen IDIs were conducted with healthcare providers comprising 15 nurse/midwives, 2 clinical officers (mid-level providers/non-physician clinicians with a 3year training), and 2 doctors, who worked in maternity, the NBU or pediatric ward in three county referral-hospitals in Homa Bay, Siaya and Vihiga Counties. In addition, IDIs with parents or caregivers (*n* = 11) of hospitalized sick newborns and very young infants (<60 days) were also conducted. Recognizing the challenges of providing critical newborn care, four themes emerged during analysis that impact on capacity and quality of newborn services in these settings (see Table 1). These four themes are: (1) facility infrastructure, medical devices and commodities for newborn care; (2) characteristics of transfers or referrals of newborns between hospitals – healthcare provider views; (3) healthcare providers' reports on caring for newborns, healthcare provider skills and competency in newborn care; and (4) Parents' experiences during transfer and hospitalization of their small or sick baby.

**Table 1:** First second and third order themes describing experiences of caring for hospitalized neonates.

Third order themes	Second order themes	Illustrative examples of experiences and reports of first order themes
1. Facility infrastructure, medical devices and commodities available in newborn units	Lack of electricity	When there is no power for long periods and there is no backup generator with fuel, the incubator goes off, the baby cannot be nursed on KMC because the mother is sick, leading to risk of hypothermia in newborn
	Lack of oxygen	Lack of portable oxygen and oxygen nasal prongs at PHC level to transfer sick baby. Providers refer babies in need of oxygen anyway hoping the baby will reach referral hospital alive.
		Provide both oxygen and incubators for referral from PHC to Hospital.
	Crowded conditions	Few incubators or cots leading to 2 or 3 babies sharing one incubator.
		Babies share incubators and may remove each other's NGT or IV-line, interrupting treatment.
		Sharing of beds/blanket by mothers and newborns in PNC
		Babies moved from incubators to KMC early as too many small babies and few incubators.
	Insufficient equipment, supplies and medicines	At PHC Providers may have neonatal skills but lack essential equipment.
		At hospitals lack of CPAP machines and sufficient incubators compromising care
		Lack of maintenance and spare parts for incubators and other equipment
		Lack of essential medicines and supplies for newborn care
	Noise disrupts newborn ability to rest	Noise from nearby construction sites.
		Squeaky NBU doors
		Frequent opening of incubators as two different babies nursed in one
2. Healthcare provider capacity	Lack of skilled staff	Not enough staff with correct knowledge and training of neonatal care at PHC level
		Sort out the staffing, training and then maybe bring equipment (incubators)
	Confidence in PHC staff	Mothers report that PHC level staff might not have required skills
	Updates for staff	People have skills, but medical skills must be improved, via continuous medical education
	Mentoring	Nurses and clinical officers at PHCs can be mentored by hospital staff. And through skills updates
	Clinical guidelines	The pediatric protocol and other guidelines available for management of the newborn
	Perinatal audit	When a neonate dies, hospitals conduct audit to understand if improvements can be made
	Teamwork	Health care providers relate well with each other
		Parents observe good communication between nurses and doctors
	Support	Parents appreciate support from providers who explain management of the baby
3. Characteristics of newborn referral - healthcare provider views	Referral from county hospital to teaching and referral hospital	Surgical or congenital conditions that need surgery because county hospitals do not have a neonatal pediatric surgeon or neonatal pediatric anesthetist.
		If mother still pregnant but in early labour for preterm delivery best to transfer her to referral hospital than wait for baby to be born and then there are challenges in referring small baby
	Limited resources at PHCs	PHC facilities have no NBU, oxygen or incubators
		Some PHCs have no staff on duty at night
	Risk of hypothermia	Newborns wrapped in cotton wool and blanket for referral
		Babies born too soon or small are at risk of hypothermia during transportation
4. Parental experience of newborn care	Family support (or lack)	Noone able to visit mother and baby due to distance and cost of transport
		Mothers are overwhelmed and stressed when they have no support
		Concern for other children at home – who is caring for them
	Friendly skilled providers/quality care	Provider from PHC drove mother and newborn to referral hospital
		Women seek care at county hospitals as health workers caring (elsewhere they harass patients)
		The nurses are very concerned about the baby
		If a hospital's services are perceived to be good by women, they believe they will get medical services quickly for their babies.
	Mothers learn new skills	Mothers are taught by healthcare providers how to care for and feed their babies.
		Mothers support and learn from each other on how to care for their babies
	Limited food available for mothers leading to sufficient milk for mothers to breastfeed	Insufficient food only 3 small meals a day
		Miss meals if attending to baby
		Mothers hungry and no funds to supplement with snacks
		Insufficient breast milk due to limited food
	Financial hardship	Loss of economic activity one to run small business
		No money
		Distance and cost of transport

### 1. Facility infrastructure, medical devices, and commodities available in newborn units

#### Overview of newborn care infrastructure in hospital

All three study hospitals have similar bed capacity for the KMC unit (8–10 beds), pediatric (3,032 beds), postnatal ward (32–35 beds) and maternity units (25–42). The NBUs in the three hospitals are placed near to the postnatal ward and mothers sleep in the postnatal ward or the KMC room while their babies are hospitalized. Critically ill or small babies are nursed in incubators or baby cots in NBU. Once a baby is stabilized (and over 1,500 g) the mother and baby would then move to the KMC unit which is also nearby or part of the NBU. There were 19–22 inpatient neonates in the NBU during data collection, with many sharing incubators or cots.

#### Medical devices and commodities

Healthcare providers from all three hospitals mentioned an inconsistent supply of medical commodities including nasogastric tubes for feeding, glucometers for measuring blood glucose levels, thermometers, oxygen, medicines, and other essential equipment and commodities.

“We should not have stock outs, when it comes to consumables like the nasal prongs for oxygen, nasogastric tubes for feeding the babies, the glucometer for doing the sugars, the “thermoguns” for taking the temperature. We should not have to experience; today I have not done this because I do not have that. That will tamper with the care that we are doing.” [IDI Vihiga Provider 3]

In addition, the availability of incubators is limited; Homa Bay and Vihiga County Hospitals and three hospitals in Siaya have incubators, lower-level facilities have none. Vihiga County Hospital has eight incubators, two are not working; Siaya has seven, and two are not working; Homabay has seven working, some with malfunctions like poor thermal regulation. This leads to sharing of incubators (2 babies in one), and the malfunctioning incubators are used as cots.

While all healthcare providers appreciated that additional smaller robust incubators would be very welcome, some providers noted however that when bringing any new medical devices, training in the basic skills of neonatal care in addition to how to use the equipment is essential.

“I would say that incubators would just be boxes if someone has no knowledge on how to use them. For example, you will have a kid in there and they have a clinician who does not even know how to calculate the feeds for this child. You first sort out the staffing, training and then maybe bring equipment (incubators).” [IDI Siaya Provider 3]

#### Equipment maintenance challenges

In addition to an inconsistent supply of electricity across all hospitals, several healthcare providers mentioned the challenges they face regarding sufficient working equipment to meet the needs of the NBU. Access to spare parts and an equipment maintenance program is essential for equipment to be kept in working order and safe to use.

“Then it [equipment] should have spare parts available. Right now, we have incubators that were given to us as donations, and they have failed, and we cannot get their spare parts. That is a challenge we have had because we have never been able to get them.” [IDI Vihiga Provider 2]

### 2. Characteristics of newborn transfer between healthcare facilities - healthcare provider views

While most newborns are transferred from PHCs to hospitals within twenty kms some are transferred to hospitals that are over 80 km away. For babies born with congenital anomalies requiring neonatologists, the journey to specialist centers can take 4–8 h. A total of 182 babies were admitted to the three NBUs (in Level 5 hospitals) in the month preceding data collection (February 2023). Healthcare providers reported that the most common reasons babies born at PHC facilities are transferred were due to preterm birth, small for gestational age, low birth weight, birth asphyxia, jaundice, respiratory distress syndrome, and sepsis.

“The number one reason for referral is prematurity; the second one is usually asphyxia when they are not able to maintain these babies on oxygen because there is no constant supply of oxygen in the peripheral facilities. The one that is also coming up is neonatal jaundice. We are receiving so many. Right now, I think we are the only place in Siaya that provides phototherapy for babies.” [IDI Siaya Provider 1]

Reasons for referral to Level 6 hospitals in Kisumu and Eldoret are usually for babies requiring neonatologists, pediatric surgeons, and anesthetists and more complex tests, scans, and surgery for congenital anomalies (such as cardiac defects, duodenal atresia, exomphalos, Hirschsprung's disease, hydrocephalus, spina bifida), babies requiring intubation, CPAP (continuous, positive airways pressure), or babies not responding to care, and requiring neonatal intensive care.

There are set criteria for transferring a baby from the Level 5 hospitals (the 3 study facilities) and is usually done by the pediatrician in consultation with the NBU team who communicates with the consultant pediatrician at the Level 6 hospital prior to moving the baby. One healthcare provider felt it was important that PHC providers had sufficient information to know where to send a baby – i.e., if a baby required surgery there is no point sending the infant to the county hospital first (Level 5) if they don't have requisite skills or specialist equipment to deal with that condition.

“And for a baby who is supposed to be referred, like for those babies who should be operated on, they do not have to refer these babies to the county hospital, knowing very well that we do not have surgeons. From the dispensary (PHC level), they can just call the Teaching and Referral Hospital directly. Coming here is a waste of time because there is nothing that is going to be done here.” [IDI Homa Bay Provider 1].

One major issue that most healthcare providers were worried about was maintaining thermal care during transfers between hospitals:

So, the transportation from PHC facilities to this level is a big challenge. During the transportation process, these babies are exposed to the cold. Sometimes they reach us while they are hypothermic and at times some may reach and survive for two days and succumb. Some might reach the hospital and even succumb on the same day. So, transportation is a great challenge. This maintenance of warmth from facility A to facility B is a challenge. [IDI Homa Bay Provider 2]

One healthcare provider mentioned that if a pregnant woman is in early preterm labor, they try to encourage staff in a PHC facility to transfer her immediately (where possible) and then she will give birth in the Level 5 hospital negating the need to transport a small sick newborn and risk getting hypothermia on the way.

“We usually say that in the event you are not able to take care of a premature baby, let the mother come while still pregnant to deliver here because transportation is usually a challenge”. (IDI Homa Bay Provider 4)

### 3. Healthcare providers’ reports on caring for newborns, provider skills and competency in newborn care

The healthcare providers (n-19) interviewed for this study were a mix of nurse/midwives (n15), clinical officers (n-2), and medical doctors (n-2), working in the maternity, NBU and or pediatric ward in one of the three study hospitals. Although student nurses may also be allocated to work in KMC and help mothers in caring for their babies, providers frequently work across the maternity, NBU and the KMC units during one shift. Staffing was sometimes challenging. For example, in one of the NBUs on the day of data collection, there were 22 inpatient neonates with just 3 staff on duty in the daytime, and at night there is often only one healthcare provider, most often a nurse, on duty.

“You can have one nurse who is supposed to be delivering, resuscitating, and looking after the babies. So staffing is a problem.” [IDI Siaya Provider 3]

#### Teamwork

Overall, there seemed to be good teamwork across the study hospitals, and staff supported each other to provide quality care. Teamwork and effective communication between mothers and staff and between different health professionals were also observed.

“Communication is key in the NBU because everything is linked…from what the nurses do, what the doctors do, physiotherapists, nutritionists…all of us are linked.” [IDI Siaya Provider 3]

“Previously we used to have babies who are less than 28 days taken to the general pediatric ward. But because of teamwork, we resolved that all babies aged under 28 days should go to the NBU.” [IDI Homa Bay Provider 4]

#### Healthcare provider skills and competency in newborn care

Most of the hospital-based healthcare providers reported the existence of national neonatal protocols, and standards of care that they had been trained in and guided their work and how to care for the small and sick newborns in the maternity/NBU. However, one area of concern was the limited skills/confidence of PHC providers, who did not have the skills to nurse small and sick newborns.

“The challenge comes when you don't know…, at first you are alone and you don't know what I am supposed to do at first, do this and this; then you don't know what you are supposed to start with, “should I start with oxygen, or should I suction first or what should I do?” Because if you do something wrong, you lose the baby. If you do not follow A, B, C, you lose the baby.” [IDI Homa Bay Provider 1].

When asked about how to improve quality of neonatal care, especially at lower-level facilities, many healthcare providers emphasized the importance of training nurses and clinical officer working at PHC level and offering regular skills updates in neonatal care prior to bringing in new or more equipment.

Not all nurses have been trained in newborn care. You see, some newborns normally go into apnoeic attacks very fast, so you must keep resuscitating and monitoring, and not many nurses have that kind of training. [IDI Siaya Provider 5]

Some of the healthcare providers interviewed act as mentors to others both within their units to update newer colleagues as well as healthcare providers working in PHC facilities. Hospitals have weekly Continuing Medical Education (CME) sessions that mentors use to update colleagues – in addition to one-to-one skills training. Key topics include the importance of preventing hypothermia, managing apnea attacks, and demonstrating the use of KMC in PHC facilities.

For example, when we are placed here in the NBU, we usually have CME trainings that we attend, to sharpen our knowledge, unlike them (lower-level facilities), who do not go for such training. [IDI Siaya Provider 6]

Quality improvement teams in each hospital are mandated to conduct perinatal and maternal mortality audits. Healthcare providers interviewed reported conducting audits to understand the causes of neonatal mortality. There were several reports of babies dying due to hypothermia during transit between hospitals and providers implied that PHC or subcounty hospital staff may not realize how important it is to keep a small baby warm in transit.

“When we are doing audits, we even call the people from the other facility to come. Because sometimes when they bring a baby who is bad, and the baby does not make it. We call them and ask where the problem started from, so that we can learn from what happened and try to teach them, and to improve on how to handle things.” [IDI Vihiga Provider 4]

### 4 Parents' experiences during transfer and hospitalization of their small and/or sick baby

Out of the 11 mothers interviewed, three gave birth in the referral hospital, six delivered at another facility and were referred, one gave birth in an ambulance, and one gave birth at home. All newborns born outside the referral hospitals arrived within one to six hours of birth, mostly via ambulance. The babies were wrapped in warm clothing but not in any specific warming device (transportable incubator) or using KMC. One mother reported that the healthcare provider drove the mother and baby to the referral hospital in his own car and helped resuscitate the baby on arrival. Mothers describe that their babies were mostly wrapped in cotton wool and warm clothing during transportation. Of the women interviewed who had babies in the NBU, there were three sets of twins (one twin died at birth), and one set of triplets. None of these women appeared to know they were expecting more than one baby. Several women said that they only started attending antenatal care after five months gestation. For most women they had other children at home. A few had previous early neonatal deaths, or miscarriages.

All mothers interviewed described similar experiences in caring for their baby. At feeding times (usually every 3 h), mothers arrive in NBU to wash their hands and proceed with feeding their baby (including naso-gastric tube feeding, cup and spoon or breastfeeding). Mothers take their babies from cots or incubators by themselves and place them back when they have finished feeding and cleaning their babies. Most of the mothers were initially taught by a nurse or they learnt from watching the nurses or other mothers and were soon confident to feed and bathe their babies and look after them in the incubators.

“In the morning at nine, I would go in and bath and give the baby milk. I would then change the towel that the baby lies on …that is what I would do in the morning. At twelve, I would also go in and give the baby milk and change the soiled diaper…just like that and I would do similar things in the evening” [IDI Siaya Parent 2].

Student nurses also assist mothers in cleaning and feeding their babies. Nurses may call a mother to soothe her baby if s/he is crying outside of feeding time. In one hospital, fathers could visit their baby in the NBU, but in other hospitals the mothers carried their babies to visit the father in the waiting room.

Researchers observed that babies in all three NBUs were exposed to persistent noise and disturbance across the three hospitals, either noise from a nearby construction site, squeaky doors or food trolleys and frequent opening and shutting of incubator doors – especially if more than one baby was nursed in each incubator – which appeared to be the case at times.

Mothers reported experiencing several similar challenges across the three hospitals: Firstly, insufficient space; they had to share a bed (and sometimes had to share one blanket) in the postnatal ward or KMC unit, and their babies had to share an incubator causing concern of possible infection from the other baby, secondly limited food available – while they all appreciated the hospital providing 3 meals a day, they all felt this was insufficient for them to establish breastfeeding. If they happened to be attending to their baby at mealtimes, they would miss food altogether and many could not afford to buy snacks or feel they could leave their baby to look for food. Thirdly, most mothers had been transferred in from other places with their newborns and lived far from their other children and support networks. Some mothers did not see any relatives at all during their stay, often due to transport costs making them feel stressed and unsupported. Several would have preferred their newborn being based in a facility nearer to where they lived, making it easier to see their other children and get support from the extended family.

“It would be better because I would be nearer home. The people at home cannot come here daily because the [bus] fare is so high. When you are nearer home, more people will be able to visit.” [IDI Siaya Parent 2]

One mother said, “*To be honest while I have been here, no one from my home has ever come to visit me here*” Which made her feel stressed and unsupported. Another mother said she had also not yet been visited by her husband, while she and her baby had been in the hospital (although they had spoken on the phone).

Finally, several mothers were worrying about economic hardship. Although they appreciated Linda Mama [national maternal health policy, which covers pregnancy care, hospital delivery, post-delivery care, and outpatient baby care] they were unable to work, and some had been in the hospital for some time.

“I have lost almost two months. I used to sell vegetables, maize and roasted maize. Now I do not have money. When I go back home, I do not even know where I am going to start from, because I am at zero.” [IDI Vihiga Parent 4]

All mothers reported receiving good quality care in the three hospitals recognizing that one of the main reasons for being there was because their babies required specialist care and nursed in incubators which were not available in the other hospitals where most of them delivered.

The treatment they [babies] get is good. Even for ones who come as emergency doctors are giving them proper attention, they are always within the vicinity of the baby, they do not sleep or sit down. [IDI Homa Bay Parent 6]

However, they all agreed that if incubators were available in more sub-county hospitals, then they would be nearer home. Although a few mothers said that the healthcare providers would also need to be skilled in caring for small and sick newborns and on how to operate incubators and other key equipment or interventions such as KMC.

## Discussion

This paper explores parent and healthcare provider experiences and perceptions on neonatal care in three county hospitals with similar characteristics in Western Kenya. The qualitative findings from this formative study describe the current experiences of healthcare providers and parents have in caring for hospitalized newborns, transport between health facilities, and discussion on how to improve quality of newborn care at lower levels of the health system.

Renewed emphasis on strategies to reduce neonatal mortality are essential to reach the third sustainable development goal (SDG), to end preventable child deaths. However, preterm newborns continue to die in LMICs because of lack of adequate care (4). Two thirds of total neonatal deaths occur during the first 3 days of life. Over 98% of deaths due to asphyxia, and 83% of deaths due to prematurity occurred in the first week after birth (17). An estimated 60 countries still need to accelerate their progress to reach the neonatal mortality SDG target of 12 deaths per 1,000 live births by 2030 (12). Neonatal deaths are preventable by known interventions delivered across the pregnancy-postnatal continuum, including interventions focusing on antenatal care, but critically during birth and the immediate postnatal period. When babies are either transported safely to or born in well-equipped facilities providing quality care, their survival is more likely (18).

However, the study hospitals have less than 10 incubators each, many not working due to unavailable spare parts. Most babies were sharing the existing incubators. The lack oxygen and other medical technologies should also not be ignored (19). One aspect described in detail by healthcare providers in this study was the number of babies referred from lower-level facilities who reached the NBU with hypothermia, with several reporting that they died on the way or shortly afterwards (9). Unlike the earlier study done in Kenya on main modes of transport for sick babies, most babies in this study were transported in ambulances (11). However, the babies were wrapped in cotton wool and coverings and not in transport incubators, nor using KMC, that would enable close observation by accompanying healthcare provider, and a stable warm environment. Thermal care is frequently neglected due to poor healthcare provider awareness (10).

Hypothermia increases the likelihood of neonatal mortality as a comorbidity parallel with other causes of newborn deaths such as sepsis, birth asphyxia and prematurity (20, 21). In Kenya, the prevalence of hypothermia may be as high as 87%, and in one study more than three quarters of newborns had hypothermia on admission, but the local data on the associated factors such as adherence to warm chain guidelines as recommended by the WHO is limited (13, 20, 21). A recent study in 21 hospitals in Kenya found that 82% of neonatal hospital admissions had their temperature recorded and of these 8,391 (17.5%) had hypothermia which represents a breakdown in warm chain protocols after birth and intra-hospital transport that can result in increased neonatal morbidity and mortality (11).

In a meta-analysis of hypothermia in neighbouring Ethiopia more than half of neonates developed hypothermia. In this study, there was a significant association between neonatal hypothermia among neonates and delayed initiation of breastfeeding, lack of skin-to-skin contact, low birth weight, and neonatal resuscitation (22).

A study in Nigeria found 5% of neonates dead on arrival at the referral hospital - where the most common modes of transport for the neonates included private vehicles (44%), and commercial vehicles – bus or taxi (41%). Other modes of transport were motorcycles (9%), by ambulance (4%), and on foot (2.5%). Babies were usually carried in the mothers or guardian's arms, wrapped in cotton wool or blankets but none used the KMC position. Only 3 babies (<1%) were transported in incubators (23). Similar findings were documented in Kenya (11).

WHO recommends standards for improving the quality of care for small and sick newborns (24). Standard 7 for small and sick newborns requires availability of competent, motivated, and empathetic staff and Standard 8 indicates that each facility has an appropriate physical environment for routine care and management of complications in small and sick newborns (24). Healthcare providers acknowledged that health workers need to be both competent and empathetic when caring for a newborn. Even working in a less than adequate environment – such as their reports of small spaces and insufficient incubators, costs etc. they were doing the best they could, given the working environment (Standard 8) (24).

However, PHC facilities lack physical space, specifically available for small and sick newborns, and rarely have sufficient staff let alone adequate competent motivated staff with neonatal skills. This raised concerns among the healthcare providers working in the NBUs interviewed for this study. Some healthcare providers with limited experience of working with neonates at PHC facilities may believe a small baby might die and so therefore do not give them due care and attention. Moreover, parents prefer for their babies to be hospitalized near home and may be reluctant to allow their baby to be referred, due to cost, cultural values and beliefs or perceptions of illness (25). One study in Eldoret, Kenya found that inappropriate thermal appliance, inadequate clothing, and late breastfeeding significantly increased the risk of neonatal hypothermia and increased risk of neonatal mortality (20, 21).

An increased focus is required on reducing the risk of neonatal hypothermia and to improve the transportation of the newborn. Rather than wrapping small newborns in layers of cotton wool and blankets making monitoring of their breathing difficult, there are two options: (1) to improve training and uptake of adopting KMC for the newborn with a parent or close relative – both at a PHC facility, or during transfer; and (2) testing a prototype of a small transportable incubator with long battery life that could be carried on someone's lap in the back of a car or placed in an ambulance or on a trolley in hospital. This would allow the healthcare provider escort to observe newborn closely in a warm stable environment. The lack of oxygen and other medical technologies should not be ignored and potentially added to any incubator design – with healthcare provider and parent input.

There appeared to be consensus among the healthcare providers interviewed that one of the most important approaches to improving newborn care was in training/mentoring of healthcare providers working in PHC facilities. Several healthcare providers mentioned that they were mentors and provided support to their colleagues using the weekly CME sessions on newborn care but felt more should be done. The mentors described how standards for transport and referral processes of sick babies between facilities were rarely followed. This is a critical area that needs increased focus. Majority of the healthcare providers interviewed were very aware of the guidelines and protocols, and competencies required, but recognized the lack of skills in those working at PHC levels.

KMC has been shown to reduce the mortality of low-birthweight newborns by a quarter when initiated immediately after birth, as well as a means for reducing hypothermia during referrals (13). The Division of Newborn and Child Health at the Ministry of Health (MoH) in Kenya promotes KMC and is in the process of adapting the WHO standards for improving the quality care for small and sick newborns (24) However PHC staff seem reluctant to support mothers to do KMC, and guidelines are not always followed to encourage bonding and to prevent hypothermia and hypoglycemia (10). Similarly, PHC providers do not seem to encourage use of KMC in an ambulance or other vehicle during referral. Although improved KMC training opportunities for healthcare providers and caregivers would help KMC uptake, there are instances when mothers are unable to do KMC or are exhausted and need a break. Additionally, the mother may be sick, in a different hospital, too far away, and no other family member is available to provide KMC. In these situations when the baby is very small or sick they will need to be in an alternative intervention to keep the baby warm –such as in an incubator, including during transfer between facilities.

Mentoring and supportive supervision, including practical skills updates in newborn care, other professional development opportunities, quality improvement teams, and clinical decision support tools all contribute to strengthened collaborative newborn care across the different healthcare levels. Moreover, supportive management including clinical audit, constructive feedback, and psychosocial support of healthcare providers creates an enabling environment (24) and have resulted in improved communication skills of providers with parents. For example, a Ugandan study demonstrated healthcare provider satisfaction and increased confidence with an easy-to-use clinical decision support software aligned with national standards of newborn care (26). In Kenya researchers introduced simple tools (job aids and videos) and a nurturing, integrative, and responsive approach for healthcare providers caring for hospitalized newborns in partnership with parents.

Consideration of parents' beliefs and practices regarding newborn disease and health outcomes and involvement in newborn health care is important (25).

Nevertheless, most mothers interviewed understood why their newborn needed the specialized care in a referral hospital where there was appropriate neonatal equipment and skilled medical and nursing staff available. There are efforts underway to expand access to appropriate neonatal equipment for use in peripheral hospitals and PHCs to address some of the challenges documented above. For example, there are currently new opportunities to expand on a co-designed collaborative process between the incubator developers and eventual users (healthcare providers and parents), to develop a sustainable low-cost small incubator model to fulfil a public good Such an incubator would build on another successful intervention to make oxygen more available in similar contexts (19). If this prototype incubator is deemed feasible and appropriate to the end users, then in the long term could potentially be manufactured in Kenya.

### Limitations

The small sample size of newborn healthcare providers (21) and mothers of newborns (9) in western Kenya may limit the generalizability of findings from these study participants. However, despite this, the detailed descriptions around newborn transfers and what happens in the NBUs have demonstrated gaps in provision of quality newborn care - including at lower level facilities in western Kenya.

## Conclusion

Hypothermia continues to be a problem for newborns – especially in PHC settings and during transfer between facilities. Findings from this study highlight potential improvements for consideration to maintain a thermal chain for small and sick newborns including more consistent use and expansion of KMC to PHC levels and during transfers. This would include a comprehensive review of healthcare provider skills on care of small and sick newborns, and updates addressed through existing MoH mentoring programs. In addition, these insights from healthcare providers and parents provide further details for product designers to co-develop or adapt and improve essential thermal equipment for newborns that is robust and appropriate for low-income settings. An increased focus on providing quality thermal care of small and sick newborns is warranted.

## Data Availability

The datasets presented in this article are not readily available because this was a qualitative study with only a few healthcare providers and parents of newborns could be identifiable. Requests to access the datasets should be directed to adriane.martinhilber@swisstph.ch.
